# Melatonin controls cell proliferation and modulates mitochondrial physiology in pancreatic stellate cells

**DOI:** 10.1007/s13105-022-00930-4

**Published:** 2022-11-05

**Authors:** Matias Estaras, Candido Ortiz-Placin, Alba Castillejo-Rufo, Miguel Fernandez-Bermejo, Gerardo Blanco, Jose M. Mateos, Daniel Vara, Pedro L. Gonzalez-Cordero, Sandra Chamizo, Diego Lopez, Adela Rojas, Isabel Jaen, Noelia de Armas, Gines M. Salido, Juan L. Iovanna, Patricia Santofimia-Castaño, Antonio Gonzalez

**Affiliations:** 1 Departamento de Fisiología, Instituto de Biomarcadores de Patologías Moleculares, Universidad de Extremadura, Avenida de Las Ciencias S/N, 10003 Cáceres, Spain; 2 Departamento de Gastroenterología, Hospital Universitario, Cáceres, Spain; 3 Unidad de Cirugía Hepatobiliopancreática Y Transplante Hepático, Hospital Universitario, Badajoz, Spain; 4 Centre de Recherche en Cancérologie de Marseille, INSERM U1068, CNRS UMR 7258, Aix-Marseille Université and Institut Paoli-Calmettes, Parc Scientifique Et Technologique de Luminy, Marseille, France

**Keywords:** Cell proliferation, Fibrosis, Glycolysis, Melatonin, Mitochondria, Pancreatic stellate cells

## Abstract

We have investigated the effects of melatonin on major pathways related with cellular proliferation and energetic metabolism in pancreatic stellate cells. In the presence of melatonin (1 mM, 100 µM, 10 µM, or 1 µM), decreases in the phosphorylation of c-Jun N-terminal kinase and of p44/42 and an increase in the phosphorylation of p38 were observed. Cell viability dropped in the presence of melatonin. A rise in the phosphorylation of AMP-activated protein kinase was detected in the presence of 1 mM and 100 µM melatonin. Treatment with 1 mM melatonin decreased the phosphorylation of protein kinase B, whereas 100 µM and 10 µM melatonin increased its phosphorylation. An increase in the generation of mitochondrial reactive oxygen species and a decrease of mitochondrial membrane potential were noted following melatonin treatment. Basal and maximal respiration, ATP production by oxidative phosphorylation, spare capacity, and proton leak dropped in the presence of melatonin. The expression of complex I of the mitochondrial respiratory chain was augmented in the presence of melatonin. Conversely, in the presence of 1 mM melatonin, decreases in the expression of mitofusins 1 and 2 were detected. The glycolysis and the glycolytic capacity were diminished in cells treated with 1 mM or 100 µM melatonin. Increases in the expression of phosphofructokinase-1 and lactate dehydrogenase were noted in cells incubated with 100 µM, 10 µM, or 1 µM melatonin. The expression of glucose transporter 1 was increased in cells incubated with 10 µM or 1 µM melatonin. Conversely, 1 mM melatonin decreased the expression of all three proteins. Our results suggest that melatonin, at pharmacological concentrations, might modulate mitochondrial physiology and energy metabolism in addition to major pathways involved in pancreatic stellate cell proliferation.

## Introduction

Pancreatic fibrosis is a condition that can impair pancreatic function. Fibrosis is a consequence of the anomalous activation of cells forming part of the stroma of the gland, together with accumulation of components of the extracellular matrix (ECM). It usually accompanies pancreatitis and/or cancer, and there is no treatment available [1]. Pancreatic stellate cells (PSCs) have been signaled as major regulators of pancreatic fibrosis. In the normal (healthy) pancreas, PSCs remain in a quiescent (resting) state and participate in the normal regulation of the extracellular surrounding. Notably, PSCs represent approximately 4–7% of the total cell population of the pancreatic tissue under physiological conditions. Nevertheless, upon damage to the gland, PSCs turn into an activated state (considered pathological) that, if not reversed, results in an increase in the proliferation of PSCs, accompanied by migration of cells, augmented deposition of ECM, and secretion of cytokines. Additionally, other inflammatory cells can infiltrate within the tissue, which enhances the deleterious conditions that are being set within the gland. As a consequence, destruction of pancreatic (either exocrine or endocrine) cells or the development of transformed cells occurs, at the time in which the healthy tissue is replaced by massive fibrosis [2]. Therefore, the control of the growth of fibrotic tissue within the pancreas is of critical relevance in the treatment of pancreatic diseases.

Melatonin (N-acetyl-5-methoxytryptamine) is an indoleamine that is produced in the mammalian pineal gland during the night. Its production follows a circadian rhythm, with high levels released into the blood at night. Melatonin receptors are widely distributed in the body, which supports that the indoleamine is able to regulate a wide array of physiological processes [3]. Moreover, evidence exist and signal that melatonin might regulate pancreatic function [4–6].

With regard to pathologies of the pancreas, melatonin has been signaled to exert possible anti-inflammatory and anticancer effects [7]. Moreover, the effects of conventional chemotherapies in different cancers were augmented in the presence of melatonin, including pancreatic cancer [8].

Of major relevance is the fact that the development of excessive fibrosis within the pancreatic gland would need to be resolved, in order to improve the response of the tissue to chemotherapy and to diminish resistance. In this line, melatonin might represent a potential therapeutical aid. Several studies, carried out on primary cultures of human and rodent PSCs, have shown that melatonin reduced the proliferation and viability of PSCs. Several mechanisms of action have been reported for melatonin to induce its antiproliferative effects on this cellular type, which include the modulation of cell cycle and of the oxidative status of the cells and activation of apoptosis [9, 10]. Nevertheless, the exact mechanisms involved in the actions of the indolamine remain to be fully elucidated. In particular, cellular energy metabolism also undergoes adaptative changes that allow fast cellular proliferation and tumor growth [11]. However, it is not entirely known whether the indoleamine might exert regulatory effects on energy metabolism in PSCs.

In this study, we aimed at providing further insights into the signaling pathways involved in melatonin actions to modulate PSC proliferation. Specifically, we analyzed whether melatonin could induce any effects on energy metabolism in PSCs, in order to clarify its potential role to control the development of fibrosis. This work is a continuation of former studies, carried out in our laboratory, to further investigate the ways by which melatonin could exert its effects on PSCs to control their proliferation and, hence, to hypothetically diminish pancreatic fibrosis.

## Materials and methods

### Chemicals

Collagenase CLSPA was obtained from Worthington Biochemical Corporation (Labclinics, Madrid, Spain). Antimycin A, Cell Lysis Reagent for cell lysis and protein solubilization, crystal violet, carbonyl cyanide m-chlorophenylhydrazone (CCCP), 2-deoxyglucose, melatonin, oligomycin, rotenone, melatonin, Tween®-20, and thapsigargin were obtained from Sigma Chemicals Co. (Madrid, Spain). Dulbecco’s modified Eagle medium (DMEM), Hanks’ balanced salt solution (HBSS), horse serum, hydrogen peroxide (H_2_O_2_), medium 199, and MitoSOX™ Red were purchased from Life Technologies (Invitrogen, Barcelona, Spain). Fetal bovine serum (FBS) was purchased from HyClone (Thermo Scientific, Erembodegen, Belgium). Penicillin/streptomycin was obtained from BioWhittaker (Lonza, Basel, Switzerland). Bradford reagent, Tris/glycine/SDS buffer (10 ×), and Tris/glycine buffer (10 ×) were from Bio-Rad (Madrid, Spain). Polystyrene plates for cell culture, SuperSignal™ West Femto Reagent, and primers for RT-qPCR were purchased from Thermo Fisher Sci. (Madrid, Spain). The list of antibodies and suppliers is given in Table 1. All other analytical grade chemicals used were obtained from Sigma Chemicals Co. (Madrid, Spain).

**Table 1:** Primary antibodies used in the study

Antibody	Dilution	Supplier
Β-Actin HRP-Conjugated	1:50,000	Thermo Fisher
p-Akt (Ser473)	1:2000	Cell Signaling
p-AMPKα (Thr172)	1:1000	Cell Signaling
p-mTOR (Ser2448)	1:1000	Cell Signaling
p-p42/44 (Thr202/Tyr204)	1:2000	Cell Signaling
p-p38 (Thr180/Tyr182)	1:1000	Cell Signaling
p-JNK (Thr183/Tyr185)	1:1000	Cell Signaling
Total OxPhos Rodent WB Antibody Cocktail	1:500	Abcam
Mitofusin 1	1:1000	Abcam
Mitofusin 2	1:1000	Cell Signaling
LAMP-1	1:1000	Abcam
Parkin	1:1000	Abcam
TIM-23	1:500	Abcam
LC3	1:1000	Cell Signaling

List of primary antibodies used for detection of the desired protein. Western blotting analysis was used, as described in the “Materials and methods” section. The corresponding secondary HRP-conjugated specific antibody was employed. Thermo Fisher (Madrid, Spain); Abcam plc (Cambridge, UK); Cell Signaling (C-Viral, Madrid, Spain)

### Preparation of cultures of pancreatic stellate cells

Primary cultures of PSCs were prepared as described previously [12]. With this procedure, cultures of activated PSCs can be prepared [9]. Pancreatic tissues were obtained from Wistar rat pups (3–5 days after birth). Animals employed in the study were supplied by the animal house of the University of Extremadura (Cáceres, Spain). Animal handling and experimental protocols were approved by the Ethical Committee for Animal Research of the University of Extremadura (reference 57/2016) and by the Institutional Committee of the Junta de Extremadura (reference 20,160,915). The experiments were carried out employing batches of cells obtained from different preparations.

### Western blot analysis

Cells were detached, centrifuged, washed with a standard phosphate-buffered saline (PBS: 137 mM NaCl, 2.7 mM KCl, 10 mM Na_2_HPO_4_, 2 mM KH_2_PO_4_; pH adjusted to 7.4), and sonicated in lysis buffer. For quantification of the protein content of samples, Bradford’s method was employed [13]. Protein lysates (20 µg/lane) were fractionated by SDS-PAGE using 10% polyacrylamide gels and transferred to nitrocellulose membranes. The membranes were incubated with the specific primary and the corresponding IgG-HRP conjugated secondary antibody. The experiments were carried out employing different batches of cells, harvested on different days.

### Determination of mitochondrial reactive oxygen species (mtROS) generation

Determination of mtROS generation was performed following previously described methods [10]. Briefly, PSCs were detached, resuspended in Na-HEPES solution 1 containing 130 mM NaCl, 4.7 mM KCl, 1.3 mM CaCl_2_, 1 mM MgCl_2_, 1.2 mM KH_2_PO_4_, 10 mM glucose, 10 mM HEPES, 0.01% trypsin inhibitor (soybean), and 0.2% bovine serum albumin (pH = 7.4 adjusted with NaOH). Next, cells were loaded with the mitochondrial ROS indicator MitoSOX™ Red for 15 min at 37 °C. Next, the cells were centrifuged (30 × *g* for 5 min) and resuspended in Na-HEPES solution 2 containing 140 mM NaCl, 4.7 mM KCl, 1 mM CaCl_2_, 1.1 mM MgCl_2_, 10 mM HEPES, and 10 mM glucose (pH adjusted to 7.4 with NaOH). Cells were then incubated with stimuli for 1 h. Generation of mitochondrial ROS was determined by measuring cellular fluorescence at 510 nm/580 nm (excitation/emission). Fluorescence was measured employing a spectrofluorimeter (CLARIOstar Plus, BMG Labtech., C-Viral, Madrid, Spain). Data show the mean increase of fluorescence expressed in percentage ± SEM (*n*) with respect to control (non-treated) cells, where *n* is the number of independent experiments.

### Determination of mitochondrial membrane potential

Mitochondrial membrane potential (*Ѱ*_m_) was recorded in PSCs loaded with TMRM. Cells were incubated in the presence of 100 nM of the dye as shown previously [14]. Fluorescence was measured employing a spectrofluorimeter (CLARIOstar Plus, BMG Labtech., C-Viral, Madrid, Spain). Results are expressed as the absolute values of fluorescence emitted at the selected excitation light, normalized to basal (pre-stimulation) fluorescence.

### XF Cell Mito Stress Test (OxPhos measurement)

PSCs were seeded at a density of 30,000 cells per well in 24-well SeaHorse® plates and allowed to attach. After 24 h, cells were in the absence or in the presence of melatonin for further 24 h. Afterwards, culture medium was replaced by DMEM culture medium without phenol red (containing 2 mM l-glutamine, 10 mM glucose, and 1 mM pyruvate). Next, cells were then incubated at 37 °C in a non-CO_2_ incubator for 1 h. Then, oxygen consumption rate (OCR) was measured under basal conditions or in the presence of 1 μM oligomycin, 1.5 µM carbonylcyanide p-(trifluoro-methoxy)phenylhydrazone (FCCP), or 0.5 µM rotenone plus 0.5 μM antimycin A. The OCR data show the absolute values normalized with respect to the number of cells, which was checked with crystal violet staining.

### XF Glycolysis Stress Test (glycolysis experiment)

PSCs were seeded at a density of 30,000 cells per well in 24-well SeaHorse® plates and allowed to attach. The next day, cells were incubated in the absence or in the presence melatonin for 24 h. Next, the culture medium was replaced by DMEM culture medium without phenol red, containing 2 mM l-glutamine. Cells were incubated at 37 °C in a non-CO_2_ incubator for 1 h. Then, extracellular acidification rate (ECAR) was measured under basal conditions and in response to 10 mM glucose, 1 μM oligomycin, and 100 mM 2-deoxyglucose (2-DG). Data show the absolute values normalized with respect to the number of cells, which was checked with crystal violet staining.

### Quantitative reverse transcription-polymerase chain reaction (RT-qPCR) analysis

RT-qPCR analysis was carried out following previously described methods [12]. In brief, cells were subjected to treatments and then lysed. Lysates were subsequently used for total RNA purification and analysis of protein. The following primers were used:

*Glut-1c*Forward: 5′-ATCCTTATTGCCCAGGTGTT-3′

Reverse: 5′-CAGAAGGGCAACAGGATACA-3′

*Ldha*Forward: 5′-GCAGGTGGTTGACAGTGCAT-3′

Reverse: 5′-ACCCGCCTAAGGTTCTTCAT-3′

*Pfkp*Forward: 5′-GACAAGATCCCCAAGAGCAA-3′

Reverse: 5′-AGCCGTCATAGATTGCGAAC-3′

*Gapdh*Forward: 5′-GGGTGTGAACCACGAGAAAT-3′

Reverse: 5′-CCTTCCACGATGCCAAAGTT-3′

The relative mRNA levels were calculated and were expressed as the fold change between the sample and calibrator (*Gapdh*).

### Cell viability assay

Determination of cell viability was carried out using the crystal violet test as described previously [15]. Briefly, after treatment, cells were fixed with 4% paraformaldehyde for 15 min at room temperature (23–25 °C) and washed with distilled water. Afterwards, the fixed cells were stained by incubation in the presence of 0.1% crystal violet (20 min at room temperature, 23–25 °C). After removal of the dye, the wells were washed with distilled water. The wells were then allowed to air dry. Next, 10% acetic acid was added to each well of the plate, followed by incubation for 20 min with shaking. In the end, 50 µL of each well was diluted 1:4 with milli-Q water and the absorbance of each sample was measured at 590 nm employing a plate reader (CLARIOstar Plus, BMG Labtech., C-Viral, Madrid, Spain). Data are shown as the mean change of absorbance expressed in percentage ± SEM (*n*) with respect to non-treated cells, where *n* is the number of independent experiments.

### Statistical analysis

Data were checked for normal distribution applying the Shapiro–Wilk test. Statistical analysis of data was performed by one-way analysis of variance (ANOVA) followed by Tukey post hoc test, and only *P* values < 0.05 were considered statistically significant. For individual comparisons and statistics between individual treatments, we employed Student’s *t* test, and only *P* values < 0.05 were considered statistically significant.

## Results

### Melatonin modulates MAPK and PI3K/Akt/mTOR signaling and reduces proliferation of PSCs

The increased proliferative rate of PSC (a classic marker of the activation process) and the increased deposition of extracellular matrix proteins are the causes of the development of the desmoplastic reaction, characteristic of pancreatic tumors [16]. Among the different pharmacological properties attributed to melatonin, antifibrotic actions have been described in different models of hepatic, renal, or pulmonary fibrosis. Our first objective was to evaluate the effect of the indolamine on PSC proliferation and viability.

**Fig. 1:**
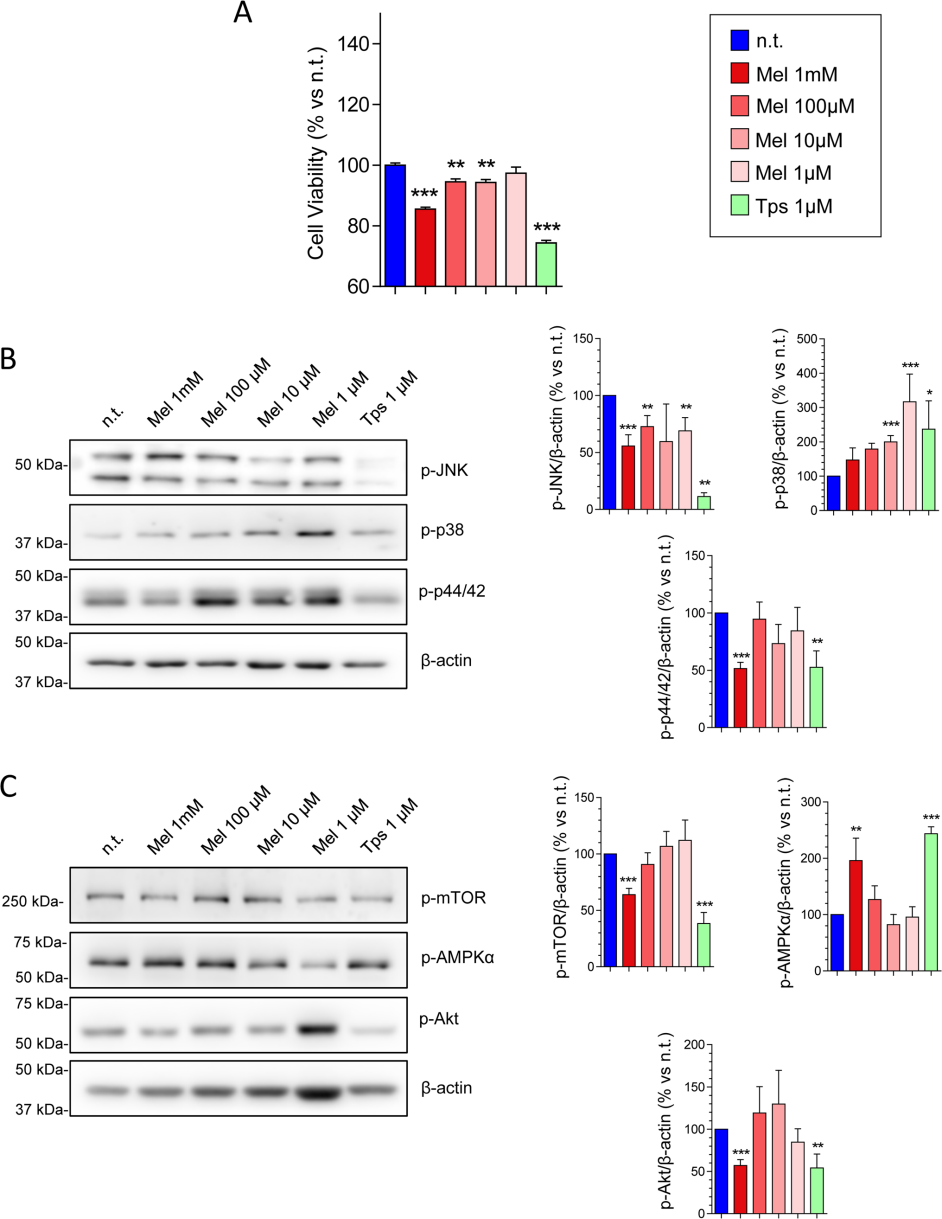
Effect of melatonin on MAPKs and PI3K/Akt/mTOR signaling and on viability of PSC. (**A**) Histogram depicting the effect of melatonin (1 mM, 100 µM, 10 µM, 1 µM) on the viability of PSC. Cells were incubated for 48 h in the absence (n.t., non-treated) or in the presence of melatonin (Mel), and cell viability was analyzed. Separate batches of cells were incubated in the presence of 1 µM thapsigargin (Tps). Results are expressed in % as the mean ± SEM (*n*) of cell viability for each treatment vs non-treated cells (incubated in the absence of melatonin). Data are representative of three independent experiments (**, *P* < 0.01; ***, *P* < 0.001 vs non-treated cells). (**B**) The blots show the level of the phosphorylated state of JNK, p38, and p44/42 in PSCs incubated for 48 h in the absence (n.t., non-treated) or in the presence of melatonin (Mel; 1 mM, 100 µM, 10 µM, 1 µM). Separate batches of cells were incubated in the presence of 1 µM Tps. The levels of β-actin were employed as controls to ensure equal loading of proteins. The bars show the quantification of protein phosphorylation for each treatment. Data show the mean ± SEM of normalized values, expressed as % with respect to non-treated cells (incubated in the absence of melatonin). Four independent experiments were carried out (Mel, melatonin; *, *P* < 0.05; **, *P* < 0.01; ***, *P* < 0.001 vs non-treated cells). (**C**) The blots show the effect of melatonin (48-h incubation) on the phosphorylation state of mTOR, AMPK, and Akt, in comparison to cells incubated in its absence (n.t., non-treated cells). The levels of β-actin were employed as controls to ensure equal loading of proteins. The bars show the quantification of protein phosphorylation for each treatment. Data show the mean ± SEM of normalized values, expressed as % with respect to non-treated cells (incubated in the absence of melatonin). Separate batches of cells were incubated in the presence of 1 µM Tps. Four independent experiments were carried out (Mel, melatonin; **, *P* < 0.01; and ***, *P* < 0.001 vs non-treated cells)

In a first step, PSCs were incubated in the absence (non-treated cells) or in the presence of melatonin (the concentrations used were 1 mM, 100 µM, 10 µM, or 1 µM) for 48 h. Separate batches of cells were incubated in the presence of 1 µM thapsigargin (Tps), a cell death inducer. After incubation of cells with the drugs, cell viability was evaluated using the crystal violet assay, which allows us to determine changes in cell number independently of metabolic activity. Viability of PSCs incubated with melatonin or with Tps was compared with that of non-treated cells, which was estimated 100% (*n* = 4 experiments). In the presence of melatonin, a decrease in cell viability was noted (Fig. 1A), which was stronger in the presence of 1 mM melatonin. In the presence of 1 µM melatonin, no changes in the viability of PSC were observed. As a control of cell death, other cells were incubated in the presence of 1 µM Tps. In the presence of this, cell viability dropped, compared with the viability of non-treated cells (Fig. 1A).

In the next step, we aimed to evaluate the status of two key intracellular signaling pathways involved in the control of cell proliferation and survival: mitogen-activated protein kinases (MAPKs) and Akt/mTOR pathways.

MAPKs are considered as major regulators of signaling pathways involved in cell stress responses, cell differentiation, cell survival, and tumorigenesis. We next studied whether melatonin could induce any effect of MAPKs in PSCs. Thus, cells were challenged with melatonin (1 mM, 100 µM, 10 µM, or 1 µM melatonin) for 48 h. As a control, other batches of PSCs were incubated in the absence of melatonin. Additionally, Tps (1 µM) was tested as a positive control of cell death in separate batches of cells. Analysis of cell lysates revealed a statistically significant decrease in the phosphorylation of JNK in PSCs incubated with melatonin (1 mM, 100 µM, 10 µM, or 1 µM), compared with the level detected in non-treated cells (incubated in the absence of melatonin). In the presence of Tps (1 µM), a statistically significant drop in the phosphorylation of JNK was observed (Fig. 1C). Treatment of PSCs with melatonin evoked increases in the phosphorylation of p38. Tps also evoked an increase in the phosphorylation of JNK (Fig. 1C). When phosphorylation of p44/42 was assayed, we observed a decrease in its phosphorylation in PSCs incubated with 1 mM melatonin, whereas no statistically significant changes were detected in cells treated with the other concentrations of melatonin. In the presence of Tps (1 µM), a statistically significant decrease in the phosphorylation of p44/42 was noted (Fig. 1C).

The Akt/AMPK/mTOR is a pivotal pathway with major roles in cell growth, survival, and proliferation. In addition, these proteins play major roles in the regulation of cellular metabolism. For these reasons, this group of proteins has been considered a key component in the development and progression of different types of cancer, including pancreatic cancer [17]. In this set of experiments, we incubated PSCs for 48 h in the presence of melatonin (1 mM, 100 µM, 10 µM, or 1 µM) or in its absence, and the phosphorylation state of several proteins of the Akt/AMPK/mTOR pathway was studied (Fig. 1C). In the presence of 1 mM melatonin, the phosphorylation of mTOR was significantly decreased. A slight decrease was detected in the presence of 100 µM melatonin, whereas no detectable changes in the phosphorylation of mTOR were noted in response to other concentrations of melatonin tested. In the presence of Tps (1 µM), a statistically significant drop in the phosphorylation of mTOR was noted. An increase in the phosphorylation of AMPK was detected in the presence of 1 mM and 100 µM melatonin. Nevertheless, no changes were noted in cells incubated in the presence of 10 µM or 1 µM melatonin. On its side, a statistically significant increase in the phosphorylation of AMPK was observed in cells treated with Tps (1 µM). Finally, a decrease in the phosphorylation of Akt was noted in cells incubated in the presence of 1 mM melatonin. On the contrary, the phosphorylation of Akt was increased in cells incubated in the presence of 100 µM and 10 µM melatonin, although the increase was not statistically significant. Treatment of PSCs with Tps (1 µM) diminished the phosphorylation of Akt.

### Melatonin induces mtROS generation, depolarization of mitochondrial membrane potential, and OxPhos impairment

Mitochondria are the major source of energy for cellular metabolism. However, these organelles are involved in many other processes such as the maintenance of redox homeostasis, Ca^2+^ signaling, the production of biosynthetic precursors, or the regulation of apoptotic processes. The actions of melatonin on mitochondria have been studied in other cancer cell models such as pancreatic tumor cells [7] or in head and neck squamous carcinoma cells. These effects have been related with the cytotoxic or antitumor actions of melatonin [18]. At this point, our next objective was to evaluate the status of mitochondria in PSCs treated with melatonin.

**Fig. 2:**
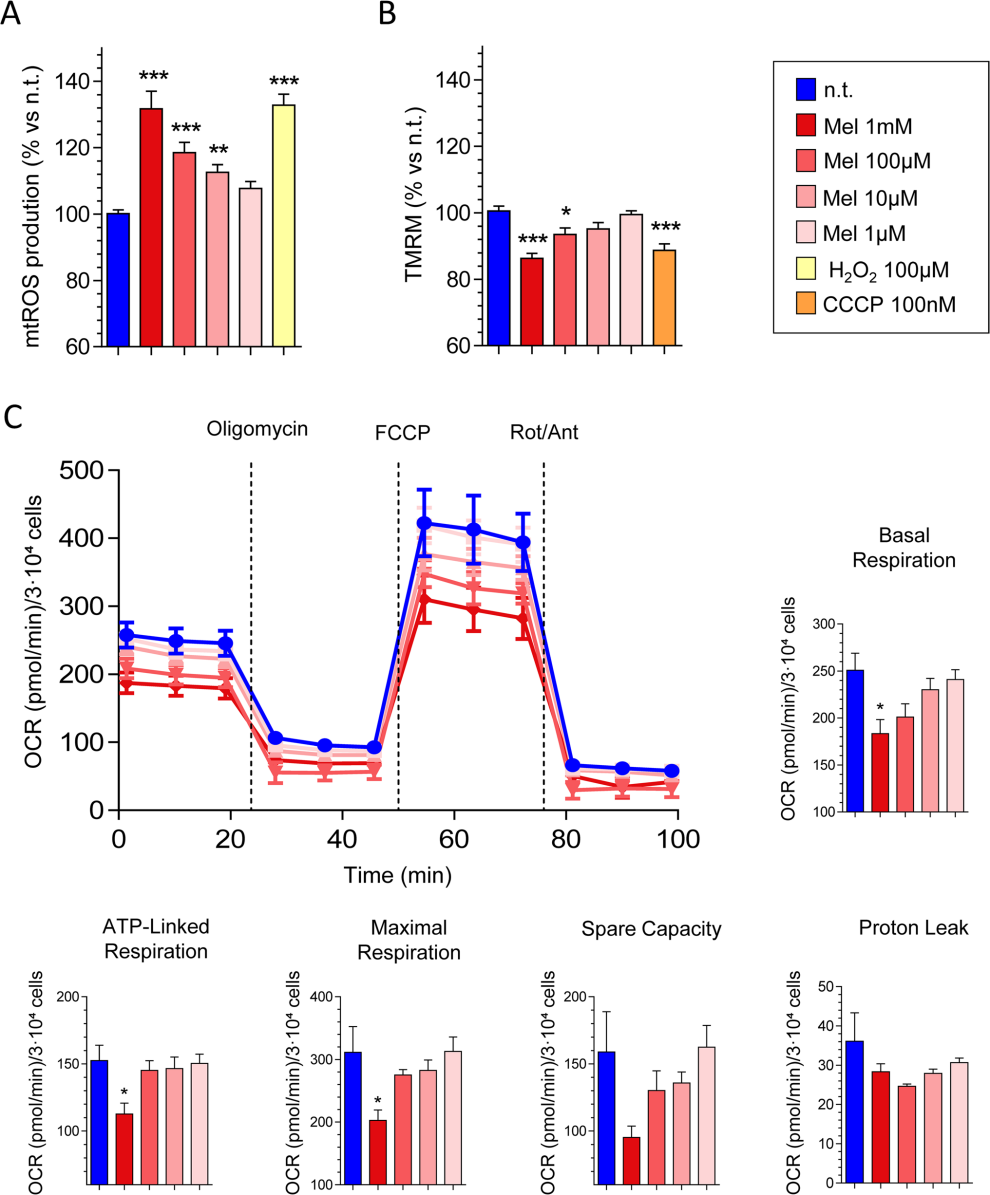
Effect of melatonin on mitochondrial reactive oxygen species generation, mitochondrial membrane potential, and on oxidative phosphorylation. (**A**) Bar chart showing the effect of melatonin on mitochondrial reactive oxygen species (mtROS; *Ѱ*_m_) generation. Separate batches of cells were incubated for 1 h in the absence (n.t., non-treated) or in the presence of melatonin (Mel; 1 mM, 100 µM, 10 µM, 1 µM) or 100 µM hydrogen peroxide (H_2_O_2_). Values are expressed in % as the mean ± SEM of cell viability for each treatment vs non-treated cells (incubated in the absence of melatonin). Data are representative of three independent experiments (**, *P* < 0.01; ***, *P* < 0.001 vs non-treated cells). (**B**) Bar chart showing the effect of melatonin on *Ѱ*_m_. PSCs were incubated for 1 h in the absence (n.t.;,non-treated) or in the presence of melatonin (Mel; 1 mM, 100 µM, 10 µM, 1 µM) or the mitochondrial uncoupler carbonyl cyanide m-chlorophenylhydrazone (CCCP; 100 nM). Values are expressed in % as the mean ± SEM of the changes in *Ѱ*_m_ for each treatment vs non-treated cells (incubated in the absence of melatonin). Data are representative of four independent experiments (TMRM, tetramethylrhodamine, methyl ester; *, *P* < 0.05; ***, *P* < 0.001 vs non-treated cells). (**C**) Cells were incubated for 24 h in the absence (non-treated, n.t.) or in the presence of melatonin (Mel; 1 mM, 100 µM, 10 µM, 1 µM). Then, oxygen consumption rate (OCR) was measured using the XF Cell Mito Stress Test Kit. The lines in the graph show the mean of OCR ± SEM of normalized values of each measurement. The OCR was measured under basal conditions or following the addition of oligomycin, carbonylcyanide p-(trifluoro-methoxy)phenylhydrazone (CCCP), or rotenone plus antimycin A (Rot/Ant). In the histograms, the bars respectively show the quantification of basal respiration, ATP-linked respiration, maximal respiration, spare capacity, and proton leak. Data were normalized with respect to the number of cells, using the crystal violet test. Results are expressed as the mean of OCR ± SEM of normalized values. Three independent experiments were carried out (*, *P* < 0.05 vs non-treated cells)

In this set of experiments, we were first interested in analyzing the effect of melatonin on the generation of reactive oxygen species within the mitochondria (mtROS). For this purpose, PSCs were incubated for 1 h in the presence of melatonin (1 mM, 100 µM, 10 µM, or 1 µM). In the presence of indoleamine, a concentration-dependent increase in mtROS generation was noted, in comparison with that detected in non-treated cells (incubated in the absence of melatonin) (Fig. 2A). Separate batches of cells were challenged with hydrogen peroxide (H_2_O_2_, 100 µM), a known oxidant. In the presence of H_2_O_2_, a statistically significant increase in the oxidation of the ROS-sensitive probe was detected.

In a next step, we tested the effect of melatonin on mitochondrial membrane potential (*Ѱ*_m_). Following addition of melatonin (1 mM, 100 µM, 10 µM, or 1 µM) to PSCs for 1 h, we observed a statistically significant decrease in TMRM-derived fluorescence, compared with the value observed in non-treated cells. The effect was more noticeable in cells incubated with the higher concentrations of melatonin tested (Fig. 2B). As a control to monitor depolarization of *Ѱ*_m_, different batches of cells were challenged with the mitochondrial uncoupler carbonyl cyanide m-chlorophenylhydrazone (CCCP; 100 nM). The compound induced a statistically significant decrease in *Ѱ*_m_, compared with that observed in non-treated cells.

Mitochondrial respiration through oxidative phosphorylation represents a major source of energy for cell function and growth, either in healthy or in pathological conditions. In this part of the research, we were interested in studying the effect of melatonin on mitochondrial respiration and its contribution to the bioenergetics status of PSCs. For this purpose, separate batches of cells were incubated in the absence (non-treated cells) or in the presence of melatonin (1 mM, 100 µM, 10 µM, or 1 µM) for 24 h. Then we analyzed several parameters related with oxidative phosphorylation. XF Cell Mito Stress revealed that melatonin decreased basal and maximal respiration, ATP production by oxidative phosphorylation, spare capacity, and proton leak. The effect was more noticeable at the concentration of 1 mM melatonin. However, no detectable changes were observed in PSCs incubated in the presence of 1 µM melatonin (Fig. 2C).

**Fig. 3:**
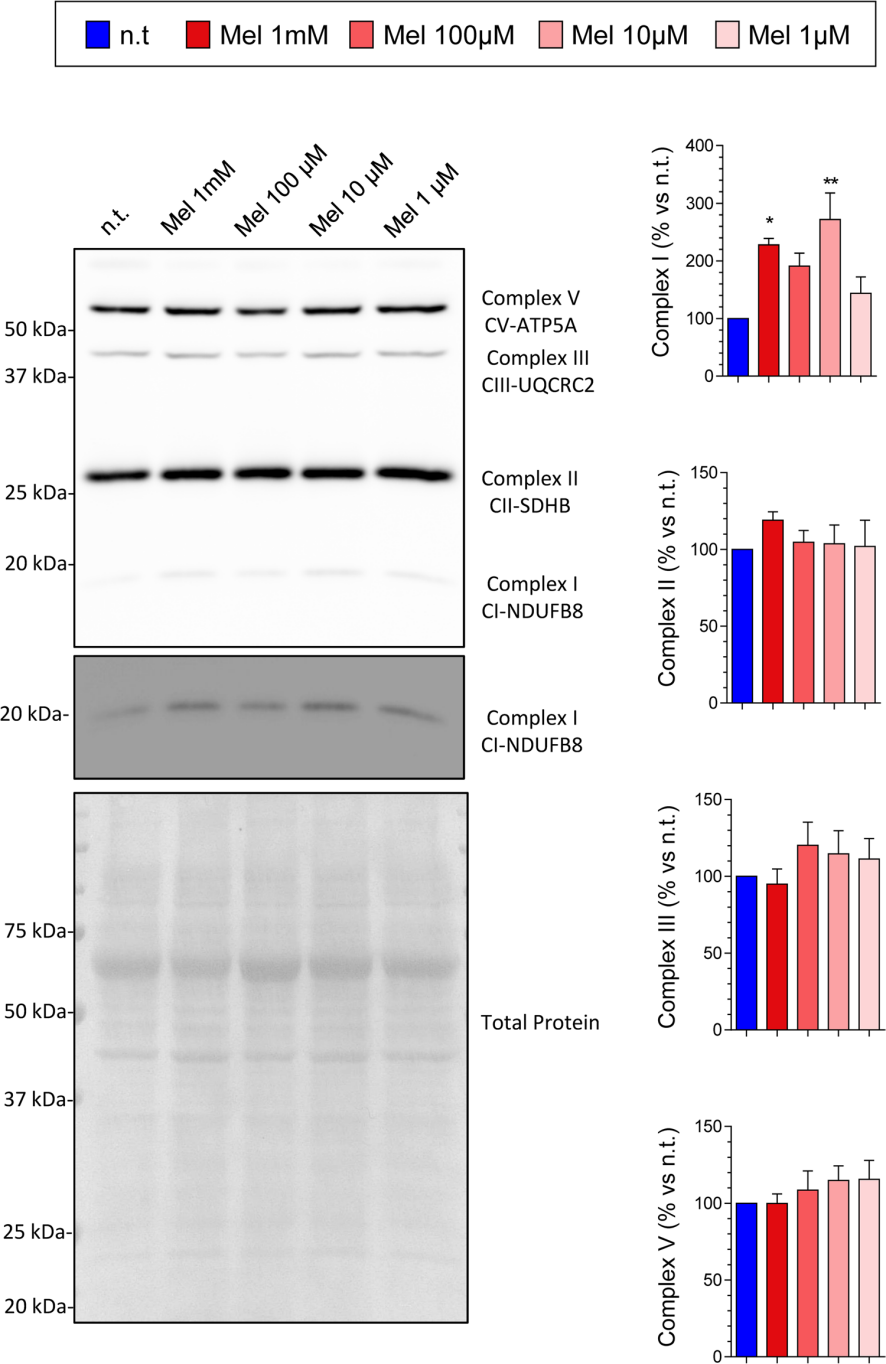
Effect of melatonin on the expression of mitochondrial respiratory chain complexes in pancreatic stellate cells. Cells were incubated for 24 h in the absence (n.t., non-treated) or in the presence of melatonin (1 mM, 100 µM, 10 µM, or 1 µM). The blots show the effect of melatonin on the level of the mitochondrial respiratory chain complexes I, II, III, and V. The levels of total protein were employed as control to ensure equal loading of proteins. The bars show the quantification of protein detection for each treatment. Data show the mean ± SEM of normalized values, expressed as % with respect to non-treated cells (incubated in the absence of melatonin). Four independent experiments were carried out (Mel, melatonin; *, *P* < 0.05; **, *P* < 0.01 vs non-treated cells)

We further studied the effect of melatonin on mitochondria in PSCs. In a next step, we analyzed the effect of the compound on the expression of mitochondrial complexes I, II, III, and V of the respiratory chain. Separate batches of cells were incubated in the absence (non-treated cells) or in the presence of melatonin (1 mM, 100 µM, 10 µM, or 1 µM) for 24 h. The effect of melatonin was different depending on the complex studied, and on the concentration applied. Following melatonin treatment, statistically significant increases in the expression of mitochondrial complex I were detected, in comparison with that noted in non-treated cells. Slight changes in the expression of complexes II, III, and V, with respect to non-treated cells, were observed, although the differences were not statistically significant (Fig. 3).

**Fig. 4:**
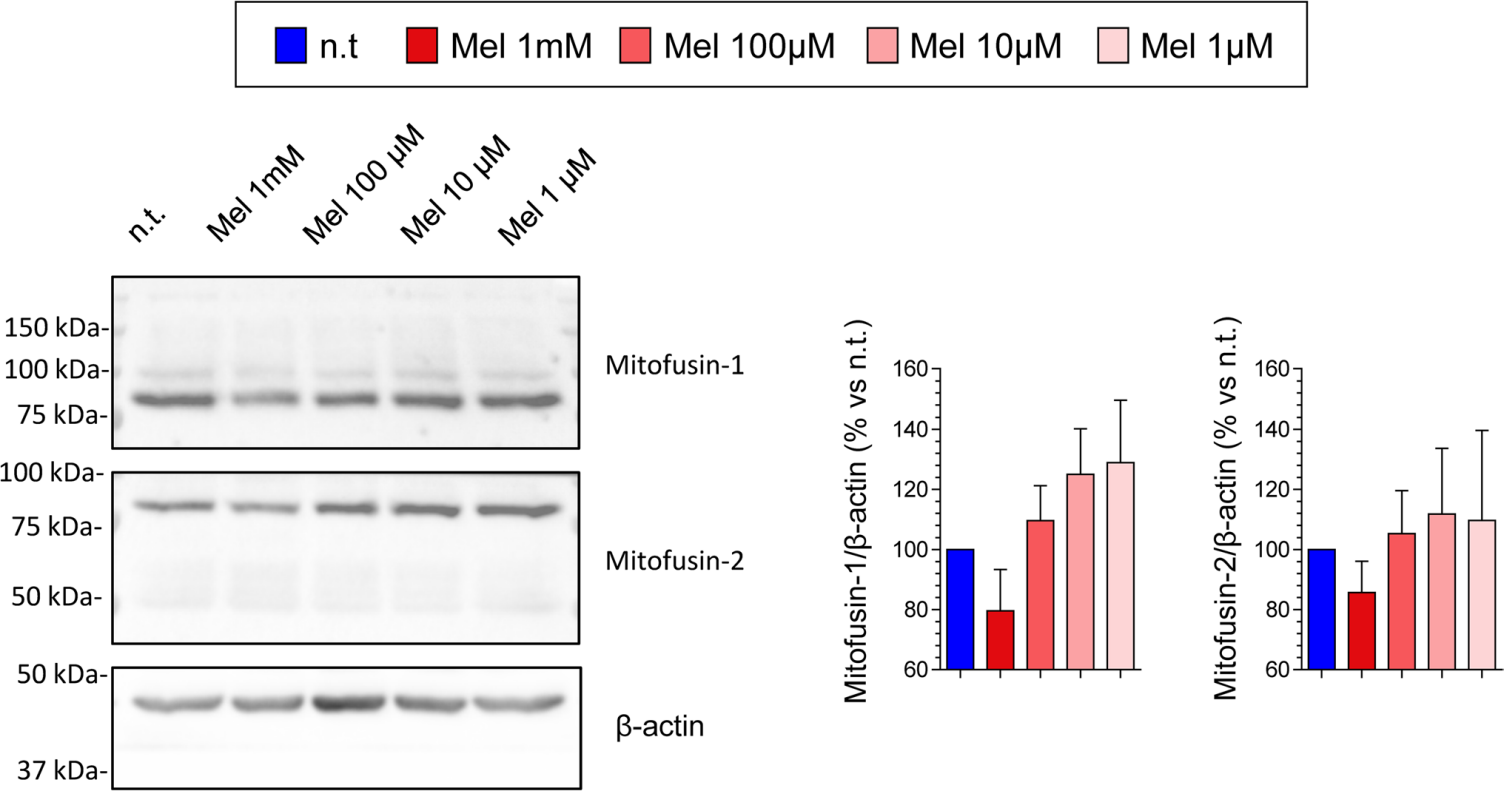
Effect of melatonin on mitofusin expression in pancreatic stellate cells. Cells were incubated for 24 h in the absence (n.t., non-treated) or in the presence of melatonin (Mel; 1 mM, 100 µM, 10 µM, or 1 µM). The blots show the effect of melatonin on the level of mitofusins 1 and 2. The levels of β-actin were employed as controls to ensure equal loading of proteins. The bars show the quantification of protein detection for each treatment. Data show the mean ± SEM of normalized values, expressed as % with respect to non-treated cells (incubated in the absence of melatonin). Four independent experiments were carried out

Fission and fusion are pivotal processes that are involved in the health of the mitochondrial network. These regulated processes represent a quality control system that removes dysfunctional organelles and are involved in cancer [19]. In this set of experiments, we were interested in analyzing the effect of melatonin on the expression of mitofusins 1 and 2. Separate batches of cells were incubated in the absence (non-treated cells) or in the presence of melatonin (1 mM, 100 µM, 10 µM, or 1 µM) for 24 h. In PSCs incubated with 1 mM melatonin, decreases in the expression of mitofusins 1 and 2 were noted in comparison with non-treated cells, although the effect was not statistically significant. The other concentrations of melatonin tested evoked slight increases in the detection of mitofusins, although the differences were not statistically significant (Fig. 4).

**Fig. 5:**
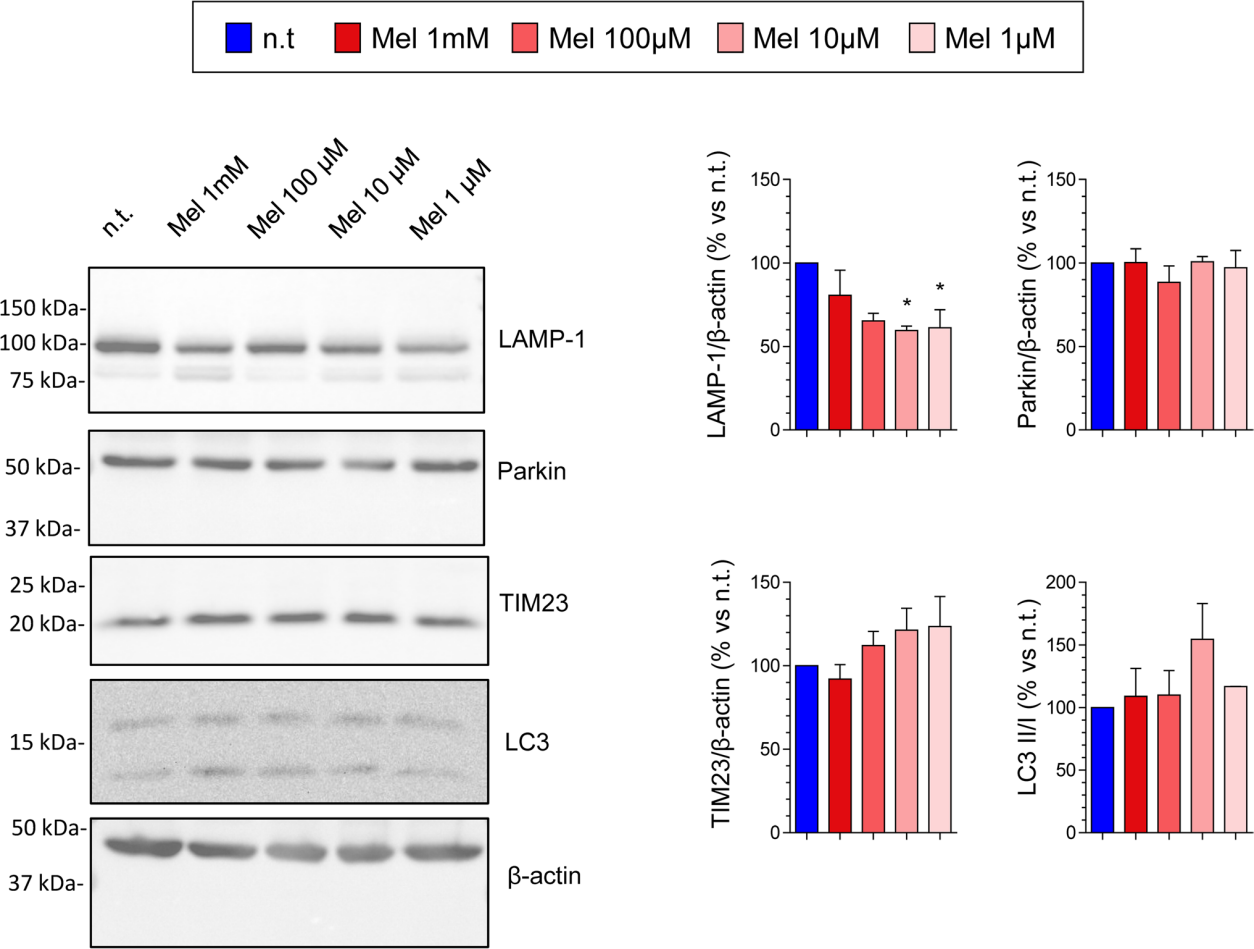
Effect of melatonin on mitophagy in pancreatic stellate cells. Cells were incubated for 24 h in the absence (n.t., non-treated) or in the presence of melatonin (Mel; 1 mM, 100 µM, 10 µM, or 1 µM). The blots show the effect of melatonin on the level of LAMP-1, Parkin, TIM-23, and LC3 I/II. The levels of β-actin were employed as controls to ensure equal loading of proteins. The bars show the quantification of protein detection for each treatment. Data show the mean ± SEM of normalized values, expressed as % with respect to non-treated cells (incubated in the absence of melatonin). Four independent experiments were carried out (Mel, melatonin; *, *P* < 0.05 vs non-treated cells)

Mitophagy is a pathway by which damaged mitochondria are destroyed and recycled [20]. Because melatonin modulates mitochondrial function and dynamics, we were interested in studying whether it could exert a regulatory role on the mitophagy process. For this purpose, we analyzed the changes of the expression of different proteins that participate in the mitophagy pathway: lysosomal-associated membrane protein 1 (LAMP-1), parkin, translocase of inner mitochondrial membrane 23 homolog B (TIM-23), and microtubule-associated protein 1A/1B-light chain 3 (LC3). PSCs were incubated for 24 h in the absence or in the presence of melatonin. Significant decreases in the expression of LAMP-1 were noted in the presence of 10 µM and 1 µM melatonin. However, we could not observe noticeable changes in the expression of the above-mentioned proteins in the presence of 1 mM melatonin, which was the concentration that had induced mitochondrial damage (Fig. 5).

### Glycolytic activity of PSC is modulated by melatonin

**Fig. 6:**
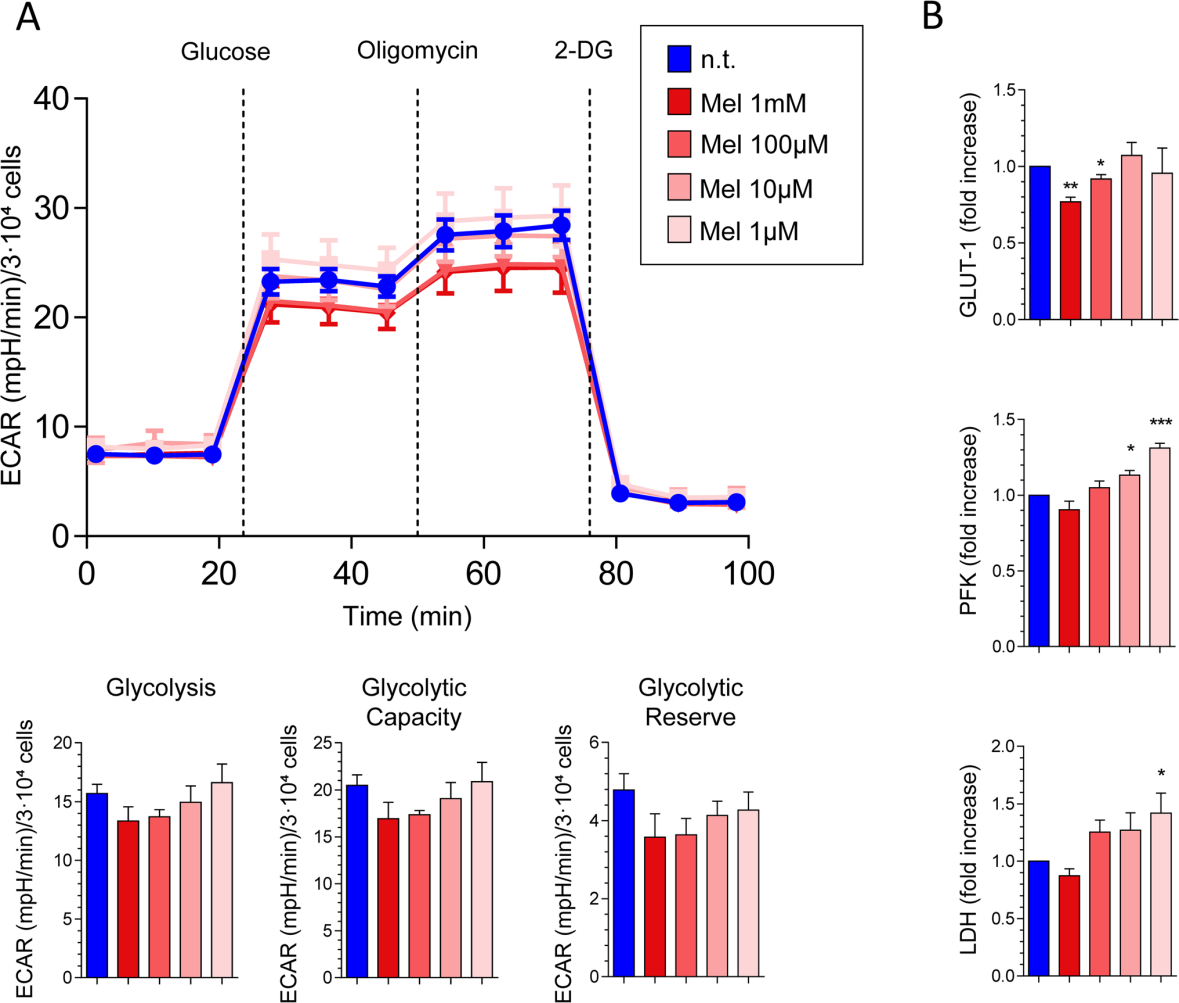
Study of glycolytic metabolism in PSCs subjected to hypoxia. Effect of melatonin. (**A**) Cells were incubated for 24 h in the absence (n.t., non-treated) or in the presence of melatonin (Mel; 1 mM, 100 µM, 10 µM, or 1 µM). Then, extracellular acidification rate (ECAR) was measured using the XF Glycolysis Stress Test Kit. The line chart shows the mean of ECAR ± SEM of normalized values of each time point measurement. The ECAR was measured under basal conditions or following the addition of glucose, oligomycin, or 2-deoxyglucose (2-DG). The bar graphs show the quantification of glycolysis, glycolytic capacity, and glycolytic reserve, respectively. ECAR data were normalized with respect to the number of cells using the crystal violet test. Results are expressed as the mean of ECAR ± SEM of normalized values. Three independent experiments were carried out. (**B**) Cells were incubated with melatonin (1 mM, 100 µM, 10 µM, or 1 µM) for 48 h in the absence (n.t., non-treated) or in the presence of melatonin (1 mM, 100 µM, 10 µM, or 1 µM) and the mRNA levels of glucose transporter 1 (GLUT-1), phosphofructokinase (PFK), and lactate dehydrogenase (LDH) were analyzed by RT-qPCR. The bars show the fold increase of mRNA levels ± SEM of each mRNA relative to cells incubated in the absence of melatonin (Mel, melatonin; *, *P* < 0.05; **, *P* < 0.01; ***, *P* < 0.001 vs non-treated cells)

As observed above, melatonin seemed to induce changes in mitochondrial activity, which might be related with energy supply to the cells. Therefore, we examined the contribution of glycolysis to cell metabolism in PSC. In this set of experiments, separate batches of cells were incubated in the absence or in the presence of melatonin (1 mM, 100 µM, 10 µM, or 1 µM). XF Glycolysis Stress Test revealed that the glycolysis and the glycolytic capacity were diminished in PSCs treated with 1 mM or 100 µM melatonin, in comparison with those detected in non-treated cells, although the differences were not statistically significant. No detectable changes were noted in cells incubated in the presence of 10 µM or 1 µM melatonin. A decrease in the glycolytic reserve was observed at all concentrations of melatonin tested, despite the differences were not statistically significant. The effect on glycolysis was more noticeable in cells incubated with the higher concentrations of melatonin tested (1 mM and 100 µM) (Fig. 6A).

In addition, we tested the effect of melatonin (1 mM, 100 µM, 10 µM, or 1 µM) on the expression of the glucose transporter 1 (Glut-1), phosphofructokinase (PFK), and lactate dehydrogenase (LDH). Therefore, PSCs were incubated with melatonin for 24 h. The effect of melatonin varied with the concentrations tested and the proteins studied. The results are shown in Fig. 6B. In comparison with the values noted in non-treated cells, we observed increases in the expression of *PFK-1* and *LDH* in cells incubated with 100 µM, 10 µM, or 1 µM melatonin. In cells treated with 10 µM or 1 µM melatonin, the expression of *Glut-1* was increased. However, a decrease in the expression of all three proteins was detected in cells incubated in the presence of 1 mM melatonin. Additionally, a drop in the expression of *Glut-1* was noted in cells incubated in the presence of 100 µM melatonin.

## Discussion

The formation of fibrotic tissue is a common pathological feature of pancreatic diseases such as inflammation and cancer. The excessive accumulation of components and cells of the extracellular matrix, at the expense of excessive secretion and/or lack of catabolism of the secreted components, impairs the balance of the normal content of the connective tissue within the gland. As a consequence, the function of the pancreas can be impaired. Moreover, the presence of growing fibrotic tissue might protect transformed cells present in the gland, which could end in the development and growth of a tumor [21]. One of the major contributors to the homeostasis of the extracellular matrix is PSC. Despite representing a low proportion of the pancreas, PSCs are considered a cell population that exhibit a critical role in health and disease because of their contribution to the preservation of the extracellular matrix and the pancreatic architecture [22]. Thus, to deal with disease, intervention might be required in order to minimize excessive deposition of extracellular components in order to prevent and/or to the successful elimination of disease [21]. Melatonin is the major product of the pineal gland and is additionally produced in other tissues and organs of the body where it could exert local effects [23]. Moreover, it can be found in vegetables and fruits and, therefore, can enter the body with diet [24]. Melatonin has exhibited antioxidant, anti-inflammatory, and anticancer effects [5]. Moreover, melatonin could be regarded as a potential antifibrotic agent, and hence as a potential therapeutical aid, provided that it has been shown to diminish the proliferation and viability of PSCs. Melatonin could induce its antiproliferative effects on PSCs through several mechanisms of action that include, among others, the cell cycle, the generation of reactive oxygen species, and apoptosis [9, 10]. Nevertheless, the exact mechanisms involved in the actions of the indolamine need to be further characterized, with particular emphasis on energy metabolism.

In this work, we have shown that, in the presence of melatonin, PSC proliferation and viability were diminished. This is in agreement with formerly reported observations [9, 10, 12]. Melatonin could involve several maneuvers to induce its effects on PSC. For example, a prooxidant effect of melatonin, which was based on the generation of ROS and a decrease in the availability of glutathione, has been observed [9, 10]. Additionally, it has been shown that melatonin induces caspase-3 activation and regulates the cell cycle through the modulation of the expression of cyclins A and D [25]. However, the molecular mechanisms involved in the antiproliferative actions of melatonin on PSCs have not been fully elucidated yet.

MAPK signaling is involved in the development of several human diseases, including neurodegenerative diseases and various types of cancers, including pancreatic cancer [26]. Our results have shown that, in the presence of melatonin, the phosphorylation of JNK and of p44/42 was diminished, whereas an increase in the phosphorylation of p38 was noted. Increased phosphorylation of p38 MAPK has been related with cell death [27]. In addition, low levels of phosphorylated JNK and p44/42 have been related with diminished cell proliferation [28]. Our results are in agreement with these previous observations and suggest that melatonin might control PSC proliferation and survival through the modulation of MAPK signaling.

On its side, the Akt/AMPK/mTOR is also a major pathway that controls cell growth, survival, and proliferation. In addition, these proteins play major roles in the regulation of cellular metabolism. An increase in the phosphorylation of AMPK and a decrease in the detection of phosphorylated mTOR have been shown in colorectal cancer cells. In addition, increases in the phosphorylation of Akt in the presence of low concentrations of melatonin and decreases in the phosphorylation of this protein in the presence of high concentrations of melatonin have been reported [29]. These previous findings support the results that we have obtained in relation with the effects of melatonin on Akt/AMPK/mTOR.

The mitochondrion has been described as one of the main sites of action of melatonin [30]. Furthermore, former results showed that melatonin induced changes in mitochondrial activity in pancreatic tumor cells that were accompanied by cell death [7]. It is well known that mitochondria are the major source of energy for cellular metabolism, which supports normal cell physiology in addition to abnormal growth of cancer cells [31]. In the present work, we observed an increase in mtROS generation and a decrease in *Ѱ*_m_ in the presence of melatonin. These results are in agreement with previous findings of our laboratory. Furthermore, our results suggest that melatonin might regulate the oxidative status of PSCs in terms of putative actions on the mitochondrial activity, as we have previously shown [7, 9, 10]. Our results further show that, in the presence of melatonin, the basal and maximal respiration, ATP production by oxidative phosphorylation, spare capacity, and proton leak were diminished. These processes are considered critical mitochondrial parameters related with energy supply to the cell. Interestingly, an increase in the expression of mitochondrial complex I was noted in the presence of melatonin. However, mitochondrial activity was diminished upon treatment of cells with the indolamine. It has been shown that the expression of mitochondrial complex I is increased in pancreatic tumors, which is accompanied by elevated mitochondrial activity. For this reason, complex I of the mitochondrial respiratory chain has been signaled as a putative therapeutic target [32]. The increase in the expression of complex I that we have observed in cells treated with melatonin could be explained as a compensatory mechanism to enhance mitochondrial activity, which could probably be targeted by melatonin.

Additionally, decreases in the expression of mitofusins 1 and 2 were noted in cells treated with melatonin. Mitofusins are located at the outer mitochondrial membrane and are involved in the control of the plasticity of mitochondria. Fission and fusion are pivotal processes that are involved in the health of the mitochondrial network and play pivotal roles in cancer [19]. Importantly, mitofusins ensure proper energetic and metabolic cellular performance [33]. Additionally, our results showed no clear changes in the expression of different proteins that participate in the mitophagy pathway. Despite melatonin treatment seems to target the mitochondria, the responses directed to dispose damaged mitochondria seem not to be activated. Altogether, these results suggest that melatonin might target mitochondrial physiology in PSC, by terms of decreased energy availability for cell proliferation. The stronger effects were noted in cells treated with the highest concentration of melatonin tested. Probably, this could be an explanation for the cytostatic effect of melatonin on PSCs.

The next set of experiments showed that the glycolysis and the glycolytic capacity were diminished in PSCs treated with melatonin. Additionally, changes in the expression of Glut-1, PFK, and LDH, which are major proteins involved in the glycolytic metabolism, were observed in the presence of melatonin. In this regard, again, the stronger effects were noted in cells treated with the highest concentration of melatonin tested. A decrease in the expression of Glut-1 in the presence of melatonin has been shown in breast cancer cells [34]. On its side, PFK depicts major roles in the metabolism of cancer cells. Thus, its modulation might represent a possible target for therapeutic intervention [35]. Finally, LDH also plays a pivotal role in cancer development. Targeting of LDH decreases cell proliferation and migration. Indeed, melatonin downregulated the activity of LDH in gastric adenocarcinoma cell line SGC7901 [36]. Our results are in agreement with all these observations and support that the antiproliferative actions of melatonin on PSC might be related with the modulation of the major regulators of energy supply to the cell.

In conclusion, our results provide evidence for the modulation by melatonin in PSC of major pathways involved in cell proliferation, like the PI3K/Akt/mTOR and MAPKs. In addition, melatonin might decrease mitochondrial activity, which was reflected by the depolarization of *Ѱ*_m_ and the generation of ROS. Interestingly, melatonin could target the complexes of the mitochondrial electron chain, which might lead to decreased energy synthesis by the organelles. Moreover, the expression of mitofusins, which are responsible for energetic and metabolic cellular performance, could be modulated by melatonin. Glycolysis could also be a point of action of melatonin. Taken together, our results provide further evidence for the mechanisms employed by melatonin to putatively modulate the physiology of PSCs, which have been signaled as major contributors to fibrosis within the pancreas.

## Abbreviations

Akt: Protein kinase B
AMPK: Activated protein kinase
2-DG: 2-Deoxyglucose
DMEM: Dulbecco’s modified Eagle medium
ECAR: Extracellular acidification rate
ECM: Extracellular matrix
FBS: Fetal bovine serum
FCCP: Carbonylcyanide p-(trifluoro-methoxy)phenylhydrazone
GAPDH: Glyceraldehyde 3-phosphate dehydrogenase
EGTA: Ethylene glycol-bis(2-aminoethylether)-N,N,N′N′-tetraacetic acid
ER: Endoplasmic reticulum
Glut-1: Glucose transporter 1
HBSS: Hanks’ balanced salt solution
H_2_O_2_: Hydrogen peroxide
JNK: C-Jun N-terminal kinase
LDH: Lactate dehydrogenase
mTOR: Mammalian target of rapamycin and mechanistic target of rapamycin
MAPKs: Mitogen-activated protein kinases
*Ѱ*_m_: Mitochondrial membrane potential
OCR: Oxygen consumption rate
OxPhos: Mitochondrial oxidative phosphorylation
PFK: Phosphofructokinase
p44/42: Extracellular signal-regulated kinase 1/2
PSCs: Pancreatic stellate cells
mtROS: Mitochondrial reactive oxygen species
RT-qPCR: Quantitative reverse transcription-polymerase chain reaction
SERCA: Sarcoendoplasmic reticulum Ca^2+^-ATPase
TME: Tumor microenvironment
TMRM: Tetramethylrhodamine
Tps: Thapsigargin
